# An Uncommon Case of Small Bowel and Pancolonic Varices

**DOI:** 10.14309/crj.0000000000000666

**Published:** 2021-10-04

**Authors:** Nicholas K. Baldwin, Sujan Ravi, Mohamed Shoreibah, Patrick S. Kamath

**Affiliations:** 1 Department of Medicine, University of Alabama at Birmingham, Birmingham, AL; 2 Division of Gastroenterology and Hepatology, University of Alabama at Birmingham, Birmingham, AL; 3 Division of Gastroenterology and Hepatology, Mayo Clinic, Rochester, MN

## CASE REPORT

**Figure 1.**
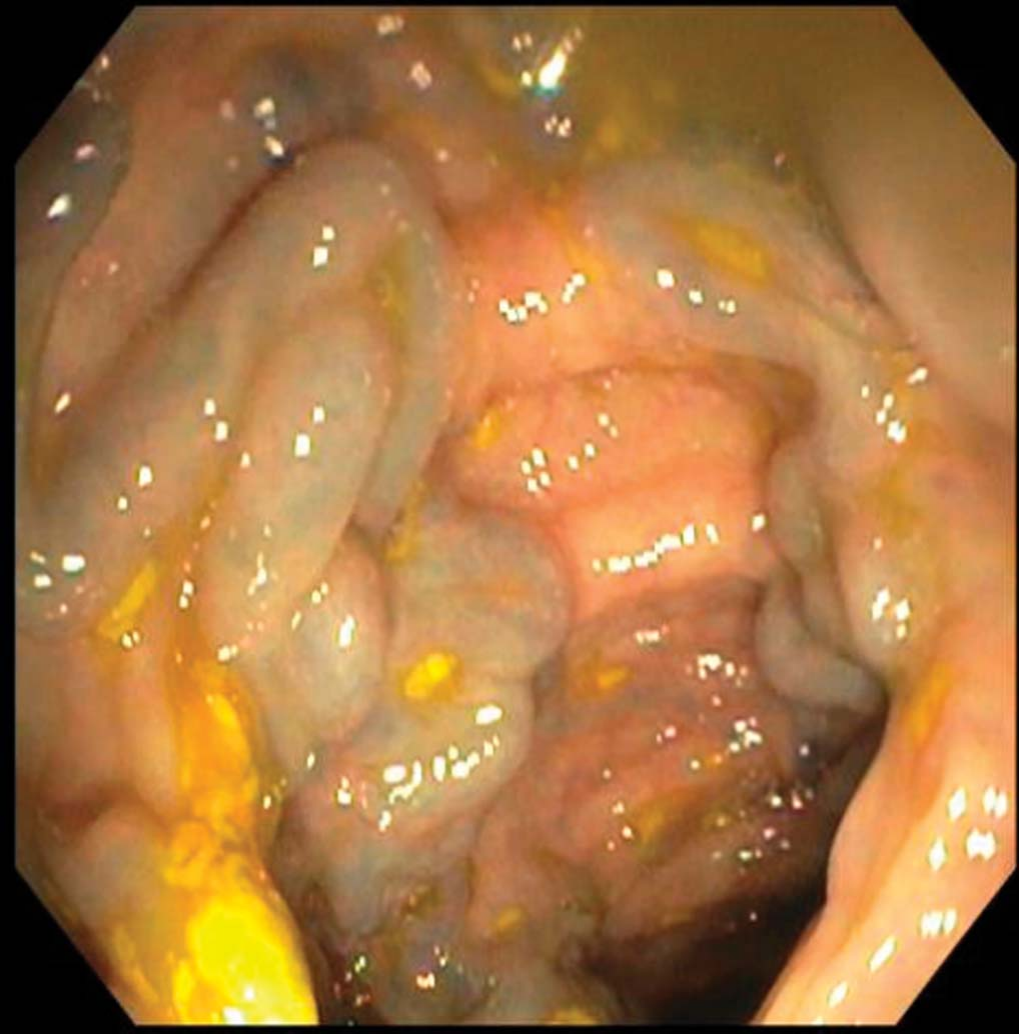
Colonoscopy revealed diffuse colonic varices extending from the cecum to the rectum.

**Figure 2.**
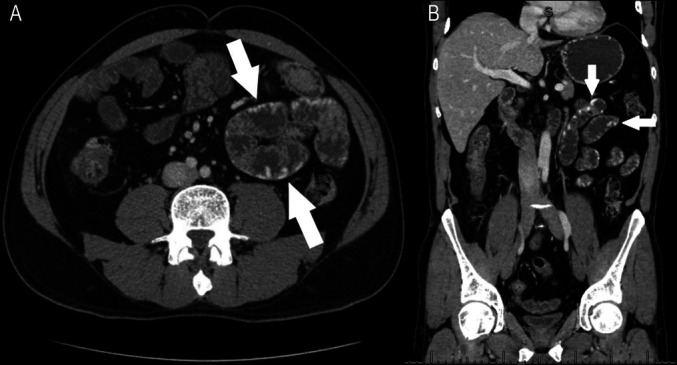
(A) Transverse and (B) coronal section of computed tomography enterography showing varices within the midjejunum (arrows).

A 41-year-old patient with no known medical history presented to clinic for evaluation of iron-deficiency anemia. The patient underwent esophagogastroduodenoscopy and colonoscopy. Esophagogastroduodenoscopy was normal with no esophageal varices or other evidence of portal hypertension. Colonoscopy revealed diffuse colonic varices extending from the cecum to the rectum (Figure 1). Computed tomography enterography showed numerous varices of the midjejunum (Figure 2). Abdominal magnetic resonance imaging showed patent portal circulation and no other abnormalities. Transthoracic echocardiogram showed no evidence of right heart failure. Transjugular liver biopsy was performed which revealed a portosytemic pressure gradient of 4 mm of Hg, indicating the absence of portal hypertension. Liver biopsy showed mild steatosis and no fibrosis. Direct measurement of portal pressure was 3 mm Hg, again indicating the absence of portal hypertension. Both the superior mesenteric vein and inferior mesenteric vein were widely patent on mesenteric angiography, with no evidence of thrombosis. In the absence of hemodynamic evidence of portal hypertension or of any portal venous obstruction, the patient was diagnosed with idiopathic small bowel and colonic varices.

Idiopathic colonic and small bowel varices in the absence of portal hypertension are exceedingly rare, with less than 40 cases reported in the literature.^1^ Most patients with idiopathic colonic varices present with massive hematochezia; however, they may be found during routine screening colonoscopy or as part of work-up for iron deficiency anemia, as was seen in this case.^2^ Colonic varices are typically the result of increased portal pressure, usually secondary to cirrhosis. The diagnosis of idiopathic colonic varices is made by excluding portal hypertension. In this case, both direct and indirect measurements of portal pressure gradient were normal, ruling out portal hypertension as a cause of the colonic and small bowel varices. Given the rarity of this clinical entity, treatment of idiopathic colonic varices remains unclear and largely depends on the patient's presentation. In elder patients and those with less significant bleeding, banding or periodic transfusions are typically performed.^3^ However, in patients presenting with massive hemorrhage or hemodynamic instability because of bleeding, surgical resection of the effected intestinal segment may be necessary to control bleeding.^4,5^ The patient described in this case was treated with oral iron supplementation with improvement in his hematocrit and serum iron stores.

## DISCLOSURES

Author contributions: NK Baldwin wrote the manuscript, revised the manuscript for intellectual content, and approved the final manuscript. S. Ravi, M. Shoreibah, and PS Kamath revised the manuscript for intellectual content and approved the final manuscript.

Financial disclosure: None to report.

Previous presentation: This case was presented at the American College of Gastroenterology Annual Scientific Meeting; October 25–30, 2019; San Antonio, Texas.

Informed consent was obtained for this case report.
